# Relations between university teachers' teaching‐related coping strategies and well‐being over time: A cross‐lagged panel analysis

**DOI:** 10.1111/bjep.12777

**Published:** 2025-04-23

**Authors:** Kristina Stockinger, Martin Daumiller, Markus Dresel

**Affiliations:** ^1^ Department of Psychology University of Augsburg Augsburg Germany

**Keywords:** coping, emotion regulation, higher education, subjective well‐being, teachers

## Abstract

**Background:**

University teachers' well‐being plays a critical role in their productivity and educational effectiveness. Apart from cross‐sectional research on demographic and institutional/contextual correlates, insight into potential causes and consequences of faculty well‐being is limited. This includes insight into relations between different coping strategies and well‐being.

**Aims:**

We studied the interplay of different strategies for coping with teaching‐related stress with university teachers' well‐being over the course of one semester.

**Sample:**

Participants were 489 German university teachers (age: *M* = 41.1 years, *SD *= 11.4) from 34 universities. Their demographics were characteristic of German university staff.

**Methods:**

Participants reported on their use of task‐oriented, emotion‐oriented and avoidance‐oriented coping to manage teaching‐related stress and on their subjective well‐being (positive and negative affect; job satisfaction) at the beginning (T1: November) and end (T2: February) of the winter 2020/2021 term. Interrelations were examined via cross‐lagged panel analysis.

**Results:**

Task‐oriented coping was positively related to the slope of changes in positive affect, and vice versa, over time. Emotion‐oriented coping (rumination) was positively related to the slope of changes in negative affect, and negatively related to the slope of changes in positive affect and job satisfaction. Negative affect was positively related to the slope of changes in avoidance‐oriented coping.

**Conclusions:**

The findings provide directions for further developing supportive measures for promoting well‐being in university teaching staff by highlighting the relevance of different coping strategies as causes and consequences thereof. Task‐oriented coping may be particularly adaptive for well‐being: at the same time, interventions aiming to promote well‐being may also facilitate task‐oriented coping behaviours.

Research has demonstrated that how individuals manage stress and emotions matters profoundly for their academic and occupational success, social functioning and well‐being; more recently, it has also indicated that well‐being, in turn, shapes individuals' capacities and selection of coping and emotion regulation strategies, implying reciprocal linkages over time (e.g., Beaumont et al., 2023; Gross, 2015; Marroquín et al., 2017; Stockinger et al., 2025). In schoolteachers, coping and emotion regulation has been linked to the quality of classroom instruction and students' learning, classroom climate and student–teacher relationship quality and teachers' own psychological and physiological well‐being (Aldrup et al., 2024; Frenzel et al., 2021; Salimzadeh et al., 2021; Wang et al., 2023). However, inquiry in teacher‐focused well‐being research is still predominantly cross‐sectional in nature (see Wang et al., 2023), providing limited insight into potential reciprocal relations between different types of regulatory behaviours and well‐being in teachers over time. This is particularly true for higher education teaching staff, a largely understudied population (Daumiller et al., 2020).

At the same time, university staff are a backbone of academic institutions and their sustainability, productivity and success in bringing forth future generations of scholars and scientists (Larson et al., 2019). As such, understanding factors that contribute to their occupational well‐being should not only be of interest in and of itself, but also for socio‐economic reasons. Against this background, the goal of the present study was to empirically verify whether previously observed bidirectional influences between coping and well‐being extend to the context of higher education teaching as well. Prior research has largely focused on demographic and contextual/institutional influences on university teaching staff members' well‐being, which seem to explain limited variance therein (Watt & Richardson, 2020). Studies addressing the role of individual characteristics and processes are needed to gain a better understanding of how to promote mental health in this occupational context.

## WELL‐BEING AND COPING WITH STRESS IN HIGHER EDUCATION TEACHING

### Subjective well‐being and stress: Core definitions

Among the many definitions of subjective well‐being (SWB) that exist in the literature (Das et al., 2020; Marsh et al., 2020), the Diener (1984) conceptualization has been widely accepted. It defines SWB as an individual's overall state of wellness that is tied to how they evaluate their lives, foregrounding the subjective nature of the construct, and emphasizes that SWB is more than the mere absence of ill‐being. It encompasses positive indicators pertaining to individuals' emotional experiences (i.e. positive/pleasant versus negative/unpleasant) as well as cognitive judgements pertaining to satisfaction with specific areas of one's life (i.e. domain satisfaction) or life globally. In this study, we focus on university teachers' occupational (i.e. domain‐specific) well‐being and measured both emotions and satisfaction related specifically to their teaching activities. High SWB implies both feeling good and functioning effectively (Huppert & So, 2013) and has been linked to better physical and mental health, more adaptive health‐related behaviours, greater educational and occupational success and higher‐quality social relationships (Bücker et al., 2018; Diener et al., 2018; DiMaria et al., 2020; Kushlev et al., 2020; Lyubomirsky et al., 2005).

From a transactional perspective, stress can be understood as ‘a particular relationship between the person and the environment that is appraised by the person as taxing or exceeding his or her resources and endangering his or her well‐being’ (Lazarus & Folkman, 1984, p. 19). It involves high‐arousal physiological responding (‘alarm reactions’; Lazarus & Folkman, 1984) that can negatively impact individuals' well‐being by predisposing them for negative affective experience, especially when they lack strategies for managing or resolving it (Scheibe & Zacher, 2013).

### Subjective well‐being in university teaching staff

Research has shown that SWB can vary across life domains (e.g. family, friends, workplace or educational settings) within individuals, and that understanding occupational SWB requires consideration of the specific challenges, affordances and demands associated with a given profession or work environment (Bakker & Demerouti, 2017; Collie et al., 2020). Accordingly, scholars have increasingly advocated for contextualized approaches to studying SWB in different occupational settings, including teaching (Bakker et al., 2007; Hascher & Waber, 2021; Sabagh et al., 2022). Studies specifically addressing university teachers' well‐being have provided increasing cause for concern and indicate a high prevalence of emotional exhaustion and diminishing job satisfaction (Daumiller & Dresel, 2020, 2023; Sabagh et al., 2018, 2022; Salimzadeh et al., 2017; Watts & Robertson, 2011). Research further indicates that institutional characteristics of academic environments and professional tasks, including teaching responsibilities, may be linked to their SWB. These characteristics include, for example, organizational climate, supportive collegiate cultures, autonomy support, provision of resources and workload (Kinman, 2014; Larson et al., 2019; Salimzadeh et al., 2017). Globally, changing occupational landscapes and increasing demands in academia, such as budget cuts, use of short‐term contracts, pressure to balance expectations for high instructional quality and research productivity in an increasingly competitive profession, fluctuations in student enrollment, increasing heterogeneity in student populations attending university, or educational reforms that may translate to considerable changes to teaching protocols have contributed to declines in well‐being and increases in stress among university instructors (Blix et al., 1994; Jayman et al., 2022; Kinman, 2014; Kinman & Johnson, 2019; Tytherleigh et al., 2005).

These developments were aggravated by the sudden and unprecedented global shift to remote teaching due to the COVID‐19 pandemic during the spring of 2020 which, on average, evidently increased teaching‐related stress (Daumiller et al., 2023; Schmidt‐Crawford et al., 2021; Schwab et al., 2022). Within this context, Daumiller et al. (2021) observed substantial interindividual differences between faculty members that were partly attributable to between‐person motivational differences, underscoring the importance of considering not only contextual but also individual factors related to university instructors' well‐being which have received considerably less attention (Watt & Richardson, 2020). Exceptions include studies linking demographic factors like gender (Blix et al., 1994; Watts & Robertson, 2011) or achievement goals (Daumiller & Dresel, 2020; Daumiller et al., 2021; Rinas et al., 2022, 2023) with subjective well‐being in university teaching staff. Individual differences in coping with teaching‐related stress are not yet well understood for this population.

### Strategies for coping with stress

Lazarus (1993) defines coping as ‘a person's ongoing efforts in thought and action to manage specific demands appraised as taxing or exceeding the resources of the person’ (p. 8). As coping shares important conceptual and functional similarities with emotion regulation, mood regulation and emotional labour in terms of both strategies and regulatory goals (Lee et al., 2016; Wang et al., 2023), it can be conceptualized as a subtype of affect regulation that targets the downregulation of negative affect and stress (Gross, 2015; Gross et al., 2019). We adopt this integrative view on different subtypes of affect regulation to review extant research and to situate our findings within the broader topically relevant literature.

Research has shown that there are substantial differences in the types of strategies individuals typically draw on to alleviate stress. The Coping Inventory for Stressful Situations (CISS‐21) developed by Endler and Parker (1990) provides a structured approach to understanding how individuals manage these stressors through distinct coping strategies: task‐oriented, emotion‐oriented and avoidance‐oriented coping. Compared with the original Ways of Coping instruments developed by Folkman and Lazarus (1980) which distinguish between emotion‐focused (i.e. regulating distressing emotions) and problem‐focused (i.e. alleviating stress by targeting its underling source) coping, the CISS‐21's additional inclusion of avoidance‐oriented coping offers a more nuanced understanding of how coping behaviours can vary. Moreover, these strategy categories align with coping behaviours reported by university staff members (Abouserie, 1996; Salimzadeh et al., 2021), underscoring their relevance for understanding coping and well‐being in this population.

Endler and Parker's (1990) conception of task‐oriented coping closely aligns with problem‐oriented coping and involves active engagement with stressors to resolve or reconceptualize stressful situations. For university teaching staff, this might include developing structured schedules to manage workload or implementing different teaching strategies to address classroom challenges. Emotion‐oriented coping involves managing emotional responses to stress and can include acceptance, relaxation strategies, rumination and excessive pre‐occupation with negative emotional experience, self‐blame, or resignation. University teaching staff might engage in this form of coping by seeking peer support to jointly dwell on frustrations during periods of academic evaluation. Avoidance‐oriented coping refers to individuals' attempts to cognitively and/or behaviourally disengage from stress‐inducing situations; according to Endler and Parker (1990, 1994), this can include distraction (e.g. situational withdrawal; shifting attention to positive/pleasant stimuli) and social diversion (e.g. seeking distraction in social engagement/consolation). In university teaching staff, avoidance‐oriented coping may involve a temporary shift in focus away from stressful academic obligations by engaging in non‐work‐related activities or social events (i.e. disengagement).

### Relations between coping and subjective well‐being

Following contemporary scholarship on coping and emotion regulation, strategies cannot be strictly categorized as *either* adaptive/functional *or* maladaptive/dysfunctional; instead, strategy use and effectiveness can vary within individuals and across situations and contexts (Stanoi & Ochsner, 2024). At the same time, individuals can differ in their habitual tendencies to rely on certain strategies, and these habits are linked to important outcomes including well‐being and social functioning (Willroth & John, 2024). Accordingly, momentary use of a given strategy may not be beneficial or harmful per se; however, findings suggest that individuals may come to develop regulatory habits that can predispose them to elevated risk of ill‐being or higher levels of well‐being, relatively speaking.

Past research corroborates the assumption that individual differences in habitual coping strategy use are linked to differences in subsequent well‐being. In general populations (Brands et al., 2014; Pisanti et al., 2014), habitual use of task‐oriented coping, which involves identifying and changing the actual source of stress, has been found to promote mental health and other favourable outcomes. Certain forms of habitual emotion‐oriented and avoidance‐oriented coping, in contrast, can impair psychological functioning and increase psychological distress, especially if used excessively over longer periods of time (see also Endler & Parker, 1994). Emotion‐oriented strategies such as rumination or self‐blame may be, on average, particularly problematic. Other strategies, such as expressive venting or emotional acceptance (emotion‐oriented coping) or disengaging via distraction (avoidance‐oriented coping), may help alleviate stress, particularly in the short term; from a long‐term perspective, however, habitual reliance on these strategies—relative to task‐oriented coping strategies—may be less effective, on average, in terms of reducing individuals' stress levels because they are not geared towards altering/resolving the actual source or problem triggering their stress.

The relation between coping and well‐being is likely not a one‐way street: Individual differences in well‐being may also impact the subsequent use of coping strategies. Theoretically speaking, affect regulation, including coping, can be conceptualized as a cyclical process in which individuals' affective experiences shape their regulatory behaviours which, in turn, influence their subsequent well‐being (Gross, 2015), implying bidirectional relations. Prior studies suggest that higher levels of subjective well‐being can promote more frequent reliance on task‐oriented coping strategies, whereas lower levels of well‐being may promote habitual reliance on emotion‐oriented self‐blame or avoidance strategies (see Freire et al., 2016, for an overview; see also Wang et al., 2024). One explanation for these patterns may be that negative affect and lower well‐being may drain individuals' cognitive and motivational resources for engaging in coping behaviours, as task‐oriented coping, which requires effort and persistence (Ford & Troy, 2019; Wang et al., 2024). Positive affect and higher levels of well‐being, in turn, may equip individuals with energetic resources and facilitate the use of task‐oriented coping strategies (Fredrickson, 2001). Taken together, coping strategies and well‐being can mutually influence each other over time, with positive feedback loops among higher levels of well‐being and (task‐oriented coping, and negative feedback loops among lower levels of well‐being and emotion‐ and avoidance‐oriented coping being plausible; see Beaumont et al., 2023, for similar theorizing and evidence).

Evidence for such feedback loops in teaching populations, especially in higher education contexts, is scant (Salimzadeh et al., 2021). Prior research on schoolteachers' coping has uncovered important findings, however, that can inform initial hypothesizing about coping and well‐being in faculty members. For instance, analyses of coping profiles in Canadian schoolteachers point to favourable emotional outcomes, on average, for individuals high in problem‐focused coping, especially in contrast to individuals high in avoidance‐focused coping (Wang et al., 2022). Moreover, a recent longitudinal investigation on schoolteachers' coping behaviours found that negative but not positive emotions predict higher average levels of subsequent avoidance‐oriented coping; however, surprisingly, coping behaviours at Time 1 did not predict subsequent experiences of emotions (neither positive nor negative; Wang & Hall, 2021). Similarly, Wang et al. (2021) found teachers' well‐being to predict their subsequent use of emotional labour strategies, but not vice versa. These findings attest to functional relevance of teachers' well‐being for coping, but fail to support systematic bidirectional relations that are typically expected based on prior theorizing and (correlational) research (Frenzel et al., 2021; Salimzadeh et al., 2021). Corresponding research addressing these mechanisms for our target population is largely lacking, to the best of our knowledge; considering that teaching contexts differ substantially between school and university settings in terms of teaching load, student age and development, and required teacher training, for example, it is important to empirically verify the generalizability of findings across occupational contexts.

## OVERVIEW OF THE PRESENT STUDY: AIMS AND HYPOTHESES

We examined relations between coping strategies and well‐being in German faculty members using a two‐wave longitudinal design. Focal variables were assessed in a contextualized manner, that is, with respect to teaching experiences during the COVID‐19‐induced lockdown over the course of one semester (T1: November 2020; T2: February 2021), providing a context that likely made teaching‐related stress and coping highly salient and thus ideal grounds for analysing reciprocal relations between coping and well‐being. We followed prior research in conceptualizing coping (task‐oriented; emotion‐oriented; avoidance‐oriented) and well‐being (positive affect; negative affect; job satisfaction) as multidimensional constructs (Endler & Parker, 1990; Rinas et al., 2023). Based on our review of prior work above, we expected initial levels of positive affect and job satisfaction to promote and thus be positively associated with the slope of changes in subsequent task‐oriented coping, and initial levels of negative affect to be positively associated with the slope of changes in emotion‐oriented and avoidance‐oriented coping. Furthermore, we expected initial levels of task‐oriented coping to be positively associated with the slope of changes in positive affect and job satisfaction, and initial levels of emotion‐ and avoidance‐oriented coping to be positively associated with the slope of changes in negative affect.

To meaningfully analyse the proposed feedback loops among coping and well‐being, we controlled for potential covariates. These included staff members' gender, as this has been found to be substantially linked to well‐being, also in university teaching staff (Blix et al., 1994; Watts & Robertson, 2011), as well as to coping (Endler & Parker, 1990, 1994). We further controlled for academic rank, levels of general and online teaching experience, perceived negative impact of the COVID‐19 pandemic on well‐being, and use of home‐office as potential covariates of well‐being.

## METHOD

### Sample and procedure

This study includes data from 489 German faculty members (*M*_age_ = 41.1 years; *SD *= 11.4; 216 female, 271 male, one non‐binary, one missing) from 34 universities with active teaching responsibilities during the COVID‐19 lockdown during the winter 2020/21 term. Participants varied in their levels of teaching experience, subject area of teaching and rank (PhD student, postdoc, professor) in a way that can be described as characteristic of German university staff based on data from the Federal Statistical Office of Germany [Statistisches Bundesamt] (2020). A detailed description of the recruitment process and sample is provided by Rinas et al. (2023), who used this dataset to examine the role of achievement goals for well‐being. Participants completed online surveys at the beginning (November 2020) and end (February 2021) of the term after providing informed consent for participating. Participants received a 5‐euro voucher and resources designed to support well‐being. This study was conducted in accordance with the ethical principles proposed by the German Psychological Society and the American Psychological Association. Ethical approval was obtained prior to data collection.

### Measures

At both measurement points, we assessed participants' coping and well‐being using established scales. Sample items and internal consistencies for all scales (all acceptable; McDonalds ω ≥ .74) are presented in Table 1.

**TABLE 1:** Sample items for coping strategies and well‐being measures as well as descriptive statistics at T1 and T2.

Scale (No. items)	Item stem and sample item	Time 1				Time 2			
		ω	Range	*M*	*SD*	ω	Range	*M*	*SD*
*Coping*									
	“When I encountered difficult, stressful, or upsetting situations in my teaching over the past month, I…:”								
Task‐oriented (7)	“…tried to analyse the problem before reacting.”	.75	1.6–5.0	3.78	0.58	.82	1.0–5.0	3.76	0.64
Emotion‐oriented (7)	“… blamed myself for being too emotional about the situation”	.86	1.0–5.0	1.95	0.81	.87	1.0–4.7	2.00	0.83
Avoidance‐oriented: Distraction (4)	“… took time off and got away from the situation.”	.70	1.0–5.0	1.85	0.81	.75	1.0–4.8	1.89	0.83
Avoidance‐oriented: Social diversion (3)	“… spent time with a friend or partner”.	.83	1.0–5.0	2.43	1.15	.74	1.0–5.0	2.23	1.07
*Subjective well‐being*									
	“Indicate the extent to which you have experienced each feeling concerning your teaching activities within the past month:”								
Positive affect (10)	“Interested.”	.86	1.0–5.0	3.49	0.66	.89	1.0–5.0	3.40	0.72
Negative affect (10)	“Distressed.”	.82	1.0–4.5	1.97	0.66	.86	1.0–4.3	1.96	0.71
Job satisfaction (4)	“All in all, I am satisfied with my role as a higher education teacher.”	.81	1.0–4.0	3.38	0.59	.84	1.0–4.0	3.34	0.65

*Note*: Theoretical ranges for coping strategies: 1–5; positive and negative affect: 1–5; job satisfaction: 1–4. Note that an extensive body of prior research using the CISS scale to measure coping has pointed to a four‐factor solution of the instrument in which avoidance‐oriented coping involves two correlated factors, distraction and social diversion, alongside the original task‐ and emotion‐oriented coping factors (for reviews and examples, see Brands et al., 2014; Pisanti, Melchiori, et al., 2015; Pisanti, van der Doef, et al., 2015).

#### Coping

Coping strategies were measured using the Coping Inventory for Stressful Situations (CISS‐21; Endler & Parker, 1990; Imran et al., 2020), which measures task−/problem‐oriented coping, emotion‐oriented coping and avoidance‐oriented coping in terms of distraction and social diversion. Items were translated into German using a back‐to‐back translation protocol involving native speakers of both languages, and the instructions were slightly adapted to fit the current research focus. Participants were asked to rate their reliance on different coping strategies to manage stressful experiences in the context of their teaching over the past month using a five‐point scale (1 = *not at all*, 5 = *very much*).

#### Well‐being

Participants rated the extent to which they experienced positive and negative emotions covered by the German version of the Positive and Negative Affect Schedule (PANAS; Breyer & Bluemke, 2016) on a scale from 1 (*very slightly or not at all*) to 5 (*very often*). Participants were asked to base their responses on their teaching experiences over the past month. Job satisfaction with teaching was measured using an adapted scale developed by the Organization for Economic Co‐operation and Development (OECD, 2014) translated into German using a translation/back‐translation protocol involving native speakers; ratings ranged from 1 (*strongly disagree*) to 4 (*strongly agree*).

#### Covariates

In addition to asking for participants' gender (male, female, non‐binary, or no answer), they reported on their academic position (doctoral student, post‐doc, professor), their general teaching experience (<1 year, 1–2 years, 2–5 years, 5–10 years 10+ years), online teaching experience, perceived negative impact of the COVID‐19 pandemic on well‐being, and use of home‐office at T1, as these aspects can reasonably be expected to relate to both use of coping strategies as well as well‐being. We dummy‐coded general teaching experience as 0 (*less than 5 years of experience*) or 1 (*more than 5 years of experience*) to distinguish between early‐career and more experienced teaching staff. Furthermore, we coded online teaching experience as 0 (*no online teaching experience prior to the semester in which the study was conducted*) or 1 (*at least some online teaching experience*), and use of home‐office as 0 (*mostly working from home*) or 1 (*mostly working in office*). Perceived negative impact of COVID‐19 on well‐being ranged from 1 (*not at all*) to 5 (*very strongly*).

### Analyses

We estimated a cross‐lagged panel model (CLPM) containing all three coping strategies and all aspects of well‐being modelled as latent variables, and controlled for gender, rank, general and online teaching experience, perceived negative impact of the COVID‐19 pandemic on well‐being, and use of home‐office (see Table S2 in supplemental materials for details on covariates). The WLSMV estimator was used to account for the categorical nature of the data, and missing data (less than 0.8% per variable; Little's (1988) MCAR test indicated to evidence of systematic missingness, *χ* ^2^(8017) = 8194.28, *p* = .081) was handled through the default WLSMV approach in Mplus (similar to pairwise deletion). All constructs were modelled as latent constructs based on the items as indicators. Following Orth et al. (2022), cross‐lagged effects of .03, .07 and .12 were interpreted as small, moderate and large, respectively.

Prior to the analyses, we checked for measurement invariance of the coping and well‐being scales following Liu et al. (2017). As institutional belonging was not a structured sampling criterion and analyses of intraclass correlations (ICCs) indicated that proportions of variance in coping strategies and well‐being attributable to this variable were small (ICCs ≤ .07), analyses were conducted at Level 1.^1^ Analyses were computed in Mplus 8.1 (Muthén & Muthén, 2017). We evaluated model fit using the comparative fit index (CFI), the Tucker‐Lewis index (TLI), the standardized root mean square residual (SRMR), and the root mean square error of approximation (RMSEA). Models with CFI/TLI ≥ .90/close to .95, and RSMEA/SRMR ≤ .10/close to .06 can be interpreted as having acceptable/good fit to the data (Hu & Bentler, 1998).

## RESULTS

### Preliminary descriptive analyses

Descriptive statistics are reported in Table 1. Faculty reported moderate to high levels of task‐oriented coping, positive affect and job and life satisfaction at both timepoints; low to moderate levels of avoidance‐oriented coping via distraction; and low levels of emotion‐oriented coping, avoidance‐oriented coping via social diversion, and negative affect at both timepoints. Almost the full theoretical range of values was observed, and there were substantial individual differences in coping and well‐being (see standard deviations). Autocorrelations across time (Table 2) showed that between one third and one half of variability in coping and well‐being T2 could be attributed to the respective values thereof at T1, leaving substantial amounts of to‐be‐explained variation.

**TABLE 2:** Bivariate manifest correlations: Coping strategies and well‐being.

	1	2	3	4	5	6	7	8	9	10	11	12	13
Coping T1													
[1] Task‐oriented													
[2] Emotion‐oriented	**−.12**												
[3] Distraction	−.09	.**30**											
[4] Social diversion	.07	.**24**	.**39**										
Well‐being T1													
[5] Positive affect	.**31**	**−.19**	−.01	.**12**									
[6] Negative affect	**−.10**	.**62**	.**28**	.**19**	**−.25**								
[7] Job satisfaction	.**20**	**−.23**	−.06	**−.10**	.**26**	**−.21**							
Coping T2													
[8] Task‐oriented	.**60**	−.08	−.07	.09	.29	−.05	.**19**						
[9] Emotion‐oriented	**−.10**	.**69**	.**25**	.**27**	**−.14**	.**48**	**−.18**	−.07					
[10] Distraction	−.06	.**25**	.**65**	.**38**	−.04	.**28**	**−.09**	−.07	.**42**				
[11] Social diversion	.08	.**19**	.**30**	.**68**	.11	.**20**	−.08	.**10**	.**29**	.**48**			
Well‐being T2													
[12] Positive affect	.**32**	**−.20**	−.01	.06	.**60**	**−.20**	.**25**	.**44**	**−.21**	−.09	.07		
[13] Negative affect	**−.14**	.**52**	.**23**	.**15**	**−.18**	.**64**	**−.16**	**−.12**	.**61**	.**35**	.**18**	**−.27**	
[14] Job satisfaction	.**19**	**−.28**	**−.09**	−.09	.**23**	**−.22**	.**74**	.**21**	**−.27**	**−.11**	−.03	.**31**	**−.28**

*Note*: *N* = 489. Boldfaced values are statistically significant at *p* < .05. Autocorrelations are highlighted in grey.

### Cross‐lagged panel model

We investigated to which extent changes in coping can be attributed to T1 well‐being, and changes in well‐being to T1 coping strategies, respectively. Prior to this, we confirmed measurement invariance. The measurement model yielded a good fit to the data (CFI = .94, TLI = .93, RMSEA = .05, SRMR = .06) and was invariant across the two timepoints (deterioration in fit from configural to metric, and from metric to scalar model: ΔCFI ≤ .001, ΔRMSEA ≤ .001, ΔSRMR ≤ .001). Fit indices for the latent cross‐lagged panel model indices were similarly good: *χ* ^2^ (4378) = 6212.59, *p* < .001, *χ* ^2^/df = 1.41, CFI = .94, TLI = .93, SRMR = .06, RMSEA = .03.

Autoregressive effects for coping strategies and well‐being indicate moderate to high stability, but also imply to be explained variation across time (*β*s ≥ .57; see Table 3).

**TABLE 3:** Standardized estimates for coping strategies and well‐being (CLPM).

Concurrent correlations	Residual correlations, T1 Coping ↔ T1 Well‐being	Residual correlations, T2 Well‐being ↔ T2 Coping
Task‐oriented coping		
Positive affect	.**43** (.05)	.**40** (.06)
Negative affect	**−.19** (.05)	−.09 (.07)
Job satisfaction	.**32** (.06)	.**22** (.07)
Emotion‐oriented coping		
Positive affect	**−.27** (.04)	**−.13** (.07)
Negative affect	.**73** (.03)	.57 (.05)
Job satisfaction	**−.20** (.06)	**−.16** (.09)
Avoidance‐oriented coping: Distraction		
Positive affect	**−.13** (.06)	**−.17** (.08)
Negative affect	.**37** (.06)	.**42** (.07)
Job satisfaction	**−.10** (.06)	**−.16** (.09)
Avoidance‐oriented coping: Social diversion		
Positive affect	.**08** (.05)	−.06 (.05)
Negative affect	.**23** (.05)	.**21** (.05)
Job satisfaction	**−.10** (.06)	.**14** (.07)

Cross‐lagged effects	T1 Coping → T2 Well‐being	T1 Well‐being → T2 Coping
Task‐oriented coping		
Positive affect	.**39** (.22)	.**11** (.05)
Negative affect	−.13 (.09)	.06 (.05)
Job satisfaction	.06 (.09)	.07 (.05)
Emotion‐oriented coping		
Positive affect	**−.25** (.15)	.03 (.04)
Negative affect	.**22** (.07)	.04 (.06)
Job satisfaction	**−.14** (.08)	−.04 (.05)
Avoidance‐oriented coping: Distraction		
Positive affect	1.31 (.98)	.05 (.04)
Negative affect	−.31 (.34)	.**14** (.06)^a^
Job satisfaction	.32 (.34)	−.07 (.05)
Avoidance‐oriented coping: Social diversion		
Positive affect	−1.18 (.91)	.05 (.04)
Negative affect	.29 (.31)	.**09** (.04)
Job satisfaction	−.26 (.31)	−.06 (.05)

*Note*: Reported are standardized parameter estimates with standard errors in parentheses. Bold coefficients. *p* < .05 (one‐tailed testing for directed hypotheses). Autoregressive paths (T1 to T2): task‐oriented coping: .64 (.04); emotion‐oriented coping: .74 (.06); avoidance‐oriented coping via distraction: .65 (.04); avoidance‐oriented coping via social diversion: .64 (.04); positive affect: .67 (.12); negative affect: .57 (.05); .83 (.04).

^a^ While this cross‐lagged effect was significant, we refrain from interpreting it due to statistical suppression: The residual covariances among T1 distraction and social diversion and correlations of changes in both variables from T1 to T2 were very high (see Table S1), resulting in suppression effects that also affected the regression weights on positive affect. To examine relations between negative affect and avoidance coping more closely, we ran two supplemental CLPMs: one including only distraction, and one including only social diversion (among the remaining coping and well‐being variables). In these models, negative affect was significantly positively related to changes in coping via social diversion but not distraction. Based on these patterns of (in‐)consistency for both coping strategies, we interpreted only the link between negative affect and social diversion.

**FIGURE 1:**
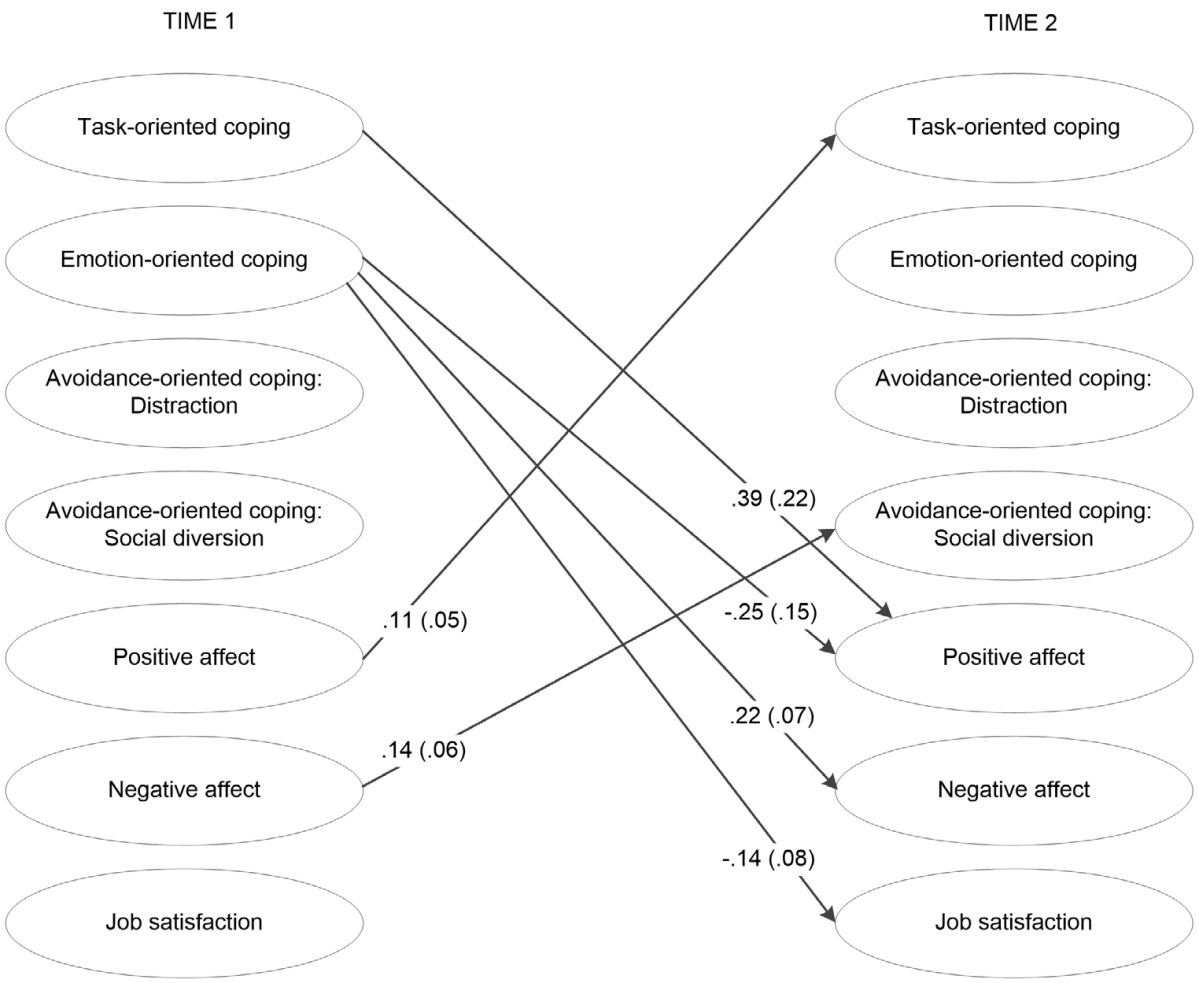
Significant cross‐lagged effects between coping strategies and well‐being. Reported are the standardized parameter estimates with standard errors in parentheses. Only paths significant at *p* < .05 (one‐tailed testing for directed hypotheses) are depicted. Gender, rank, general teaching experience, online teaching experience, perceived negative impact of the COVID‐19 pandemic on well‐being, and use of home‐office were included as control variables. All autoregressive effects were significant, ranging from .57 to .74 (see Table 3 for full results of all parameters). Residual correlations between coping strategies and well‐being were modelled at T1 and T2, respectively, and are not depicted for clarity.

As summarized in Figure 1, T1 task‐oriented coping was positively related to the slope of changes in positive affect, and T1 positive affect was positively related to the slope of changes in task‐oriented coping, implying positive feedback loops among these constructs over time, as expected.

Emotion‐oriented coping was positively related to the slope of changes in negative affect, as hypothesized; however, it was also negatively related to changes in positive affect and job satisfaction. Further in line with our hypotheses, initial levels of negative affect were positively related to the slope of changes in avoidance‐oriented coping via distraction and social diversion. For the remaining coping strategies and well‐being indicators, no statistically significant relations over time emerged (Table 3; see Tables S1 and S2 for additional information on residual correlations, concurrent correlations and effects of covariates).

## DISCUSSION

### Summary and interpretation of findings

This study examined relations between university teaching staff members' strategies for coping with teaching‐related stress and their occupational well‐being over time, spanning one semester of remote teaching during the COVID‐19 pandemic. Among the multitude of tasks and adversities this occupational group faces—such as providing instruction on top of balancing research and administrative duties and enduring short‐term contracts and intense competition—teaching, particularly under pandemic conditions, can be a major source of stress for university teaching staff. This is especially true given that they are expected to deliver high‐quality instruction, often with little or no formal training.

Our findings attest to the assumption that faculty members' strategies for coping with teaching‐related stress can impact their well‐being over time, and vice versa – in other words, how they feel matters for their approaches to coping, and these, in turn, matter for subsequent well‐being. Corroborating our hypotheses, we found moderate to large positive reciprocal relations between task‐oriented coping and positive affect related to teaching. These findings align with prior research that documents the psychological benefits of task‐oriented coping, in which individuals actively resolve their stress by targeting its origins (Brands et al., 2014; Endler & Parker, 1994; Pisanti et al., 2014), and highlight that higher levels of well‐being can indeed facilitate the adoption of such coping strategies. Accordingly, given the observed bidirectionality of these relations, interventions aimed at fostering well‐being—for instance, by way of supporting perceived autonomy (Dreer, 2023)—may serve as a protective measure in terms of helping instructors adopt more adaptive patterns of habitual coping via active problem solving that can help maintain or boost subsequent well‐being.

We also found support for the expected negative consequences of emotion‐oriented coping which relies heavily on rumination, excessive worrying, self‐blame and resignation, on subsequent levels of negative but also positive affect as well as job satisfaction (large effects). Again, these findings are largely aligned with prior research highlighting that habitual reliance on such coping strategies can compromise individuals' well‐being and health (Brands et al., 2014; Endler & Parker, 1994; Pisanti et al., 2014). However, we did not find evidence for cross‐lagged effects of negative affect on subsequent use of emotion‐oriented coping as reported in prior work (Kariv & Heiman, 2005); this is partly due to the relatively high stability of emotion‐oriented coping over the timespan considered. Moreover, such cross‐lagged effects are likely smaller and may require different methodological approaches, such as high‐frequency experience sampling designs that capture momentary use of coping strategies, to be detected.

Instead, negative affect was positively related to changes in reliance on social diversion (large effect), partially corroborating our hypotheses. Consistent with the stress‐buffering hypotheses (Cohen & Wills, 1985), for example, this finding suggests that university teaching staff experiencing higher levels of negative affect turn to social connections to disengage from stress. However, neither distraction nor social diversion was related to subsequent changes in well‐being, neither positively nor negatively. Avoidance‐oriented strategies are typically listed among the putatively maladaptive strategies linked to increases in ill‐being (e.g., Naragon‐Gainey et al., 2017); however, select studies have reported zero relations with subjective well‐being (e.g. Fischer et al., 2021). Based on our findings, engaging in social diversion to cope with teaching‐related stress may not be recommendable because, on average, this strategy does not seem to help reduce negative affect, likely because it essentially prohibits individuals from resolving the underlying cause of their stress. Generally, the effects of avoidance‐based approaches to coping on well‐being may also be more variable across situations and their specific enactment, but more research on mechanisms driving these linkages is needed.

Overall, these patterns point to the importance of taking a closer look at both the functional impact of different types of strategies available for realizing broader coping styles, as well as further factors that shape university teaching staff members' tendencies to rely on certain types of strategies over others (Willroth & John, 2024). Our data generally revealed stronger and more frequent linkages between prior levels of coping strategy use and subsequent levels of well‐being than vice versa. Moreover, these linkages were more pronounced for positive and negative affect as compared with job satisfaction. This might be due to the cognitive‐evaluative nature of this construct (Diener et al., 2018), while coping strategies target affective experience. However, to some extent, these patterns may also be attributable to the high stability of the variable across timepoints. We thus reiterate the call for more research on relations between coping and well‐being over time based on longitudinal designs with multiple assessment waves which afford multiple lags for analysing the directionality of interrelations and for disentangling inter‐ and intraindividual processes based on different time spans (Rinas et al., 2023; Wang & Hall, 2021).

While our hypotheses were only partially supported, our findings speak to especially strong reciprocal ties between task‐oriented coping and positive affect. That we found this rather isolated cross‐lagged effect might also be a function of the research design with two measurement points. However, the bivariate correlations between task‐oriented coping and both emotion‐ and avoidance‐oriented coping, as well as between positive affect, negative affect and job satisfaction, were only small to moderate, pointing to an important, reinforcing feedback loop.

### Limitations and directions for future research

Several limitations need to be considered when interpreting our findings and deriving directions for future research.

First, we focused on three major categories of coping strategies along the conceptualization by Endler and Parker (Endler & Parker, 1990; see Daumiller et al., 2024, for a similar classification of emotion regulation strategies). These represent a higher‐level functional organization in terms of broader strategy families that can be enacted through various tactics (Gross, 2015). It is possible that different ways of enacting these broader strategies can impact well‐being in different ways (see, e.g., Stockinger et al., 2025, a, for evidence on differential relations between emotion regulation strategies and student health and well‐being). Accordingly, we recommend future work to complement the present findings by considering a broader array of self‐regulatory strategies instructors may have at their disposal to manage emotional challenges related to their teaching (Taxer & Gross, 2018). Furthermore, in line with recent findings documenting the importance of considering not only frequency of regulatory strategy use, but also the situation‐specific fit of chosen strategies as well as the quality of strategy application (including, e.g. planning, monitoring and adapting strategy use if needed; see, e.g. von der Mülbe et al., 2024), for gaining deeper insight into the functions and relative effectiveness of different approaches to coping with stress.

Second, while our sample demographics were characteristic of German university staff throughout the country with regard to gender, age and academic ranks, our findings are naturally restricted to this particular socio‐cultural context and thus to be interpreted as such. Future research should probe the generalizability of the observed feedback loops among task‐oriented coping and well‐being in other countries and across different higher education systems that may also entail different academic support systems. Similarly, future studies should attend to different sources of stress encountered by academic staff at university, including research‐related pressures or funding/job insecurities, to gain a fuller understanding of relevant stressors in this occupational group and to verify to which degree relations between coping strategies and well‐being generalize across them.

Third, the study was conducted during the unique circumstances of the COVID‐19 pandemic, when university teaching staff had to quickly adapt their traditional teaching practices to online formats, posing significant challenges for many. While this context posed significant threats to well‐being and necessitated coping mechanisms, it also means that some effect sizes may have been overestimated. Accordingly, this warrants further replication of our findings in less demanding situations.

Fourth, we focused on subjective well‐being, which, like the use of coping strategies, was assessed via self‐report. While the longitudinal approach may have mitigated some potential biases inherent to self‐reports due to the time between both measurement points (Jordan & Troth, 2020), this concern cannot be entirely ruled out. While self‐reports are considered standard approaches for the assessments of the constructs at hand (with subjective well‐being being subjective, by definition and coping focusing on largely covert behaviours), and alternatives for their assessments have not been established, future research could consider additional indicators of well‐being and health, such as physical health indicators like psychosomatic stress symptoms, health behaviours, or metrics such as the number of sick days.

Relatedly, our assessments focused on university teaching staff members' behaviour in the past month. Such an assessment might be prone to retrospective biases. Accordingly, situated/momentary assessments of coping and well‐being using experience sampling may be particularly suitable for capturing dynamic fluctuations in these constructs (McMahon & Naragon‐Gainey, 2018) and for studying variability in situation‐specific effects of different regulatory strategies for different individuals (i.e. what works, for whom, under which conditions). Employing experience sampling or diary methods would allow for the collection of more immediate data on these constructs within the everyday context of university instructors' lives, thus also enhancing the ecological validity of the results (Csikszentmihalyi & Larson, 2014; Sandvik et al., 2009). Additionally, designs covering multiple assessment waves allow for analysing reciprocal relations between coping and well‐being at the within‐person level (i.e. using random‐intercept CLPM; Hamaker et al., 2015), thus complementing the present study which focuses on between‐person relations over two timepoints and providing insight into the degree to which the observed relations hold both between and within individuals (see Beaumont et al., 2023, for a similar approach and evidence for convergence between emotion regulation strategy use and student well‐being over time).

### Practical implications and conclusion

This study explored possible mutual influences of different coping strategies on university teaching staff members' well‐being, and vice versa, over one semester of teaching. The positive, bidirectional connections over time between task‐oriented coping and positive affect indicate that occupational well‐being is not only a product, but also condition for engaging in productive coping; thus, they underscore the importance of systematically *combining* extant approaches for fostering university teachers' occupational well‐being including, for example, mindfulness‐based approaches (see Avola et al., 2025; Herman et al., 2020) with trainings that specifically target individuals' knowledge and use of different types of coping strategies. Our findings suggest that fostering *task‐oriented coping* may be particularly beneficial for well‐being; at the same time, preventing negative consequences of rumination‐focused emotion‐oriented coping on well‐being seems warranted.

Specifically, academic support systems could integrate psychoeducation on coping and well‐being directly in workshops and counselling. This support could include three key components (see also Avola et al., 2025; Herman et al., 2020). First, discussing different ways of coping and boosting broad, flexible strategy repertoires (e.g. Aldao et al., 2015) that include task‐oriented regulatory strategies. Second, engaging faculty in reflective exercises to determine how they have used such strategies previously and how effective they were. Third, offering practical training on situationally fitting as well as effective (i.e. high quality) strategy implementation. However, to effectively guide such interventions, further research based on the present findings is necessary. Gaining a better understanding of the processes underlying adaptive regulation in faculty members is a critical endeavour for ensuring individual thriving as well as educational quality in higher education institutions.

## AUTHOR CONTRIBUTIONS

**Kristina Stockinger:** Formal analysis; writing – original draft; writing – review and editing; conceptualization; methodology. **Martin Daumiller:** Conceptualization; methodology; formal analysis; writing – review and editing; project administration; data curation. **Markus Dresel:** Conceptualization; funding acquisition; methodology; writing – review and editing.

## FUNDING INFORMATION

The research reported in this article was supported by the German Federal Ministry of Education and Research, grant no. DR 454/8‐1 awarded to Markus Dresel.

## CONFLICT OF INTEREST STATEMENT

We have no known conflict of interest to disclose.

## Supporting information


Table S1.
Table S2.

## Data Availability

The data that support the findings of this study are available from the corresponding author upon reasonable request.

## Footnotes

^1^
We also re‐ran our CLPM analyses using the ‘type = complex’ command. The results remained substantively unchanged, confirming that institutional belonging did not systematically influence our findings.
