# Supplementary material for: Relations between university teachers' teaching‐related coping strategies and well‐being over time: A cross‐lagged panel analysis
Source: Br J Educ Psychol. 2025 Apr 23;96(1):54–72. doi: 10.1111/bjep.12777 (PMC12879523; doi:10.1111/bjep.12777)
Supplement: Supplementary file 1 — Table S1. Table S2. [file BJEP-96-54-s001.docx]

Supplemental Material for

**Relations between University Teachers’ Teaching-Related Coping Strategies and Well-Being Over Time: A Cross-Lagged Panel Analysis**

This file includes Supplemental Tables S1 and S2.

**Table S1**

*Residual Correlations and T1-T2 Change Correlations among Coping Strategies and Well-Being Facets (Cross-Lagged Panel Model)*

| Variable set | Residual correlations  T1 ↔T1 | Correlations of changes  T1-T2 ↔ T1-T2 |
| --- | --- | --- |
| Coping strategies  Task- \| Emotion-oriented | **–.21** (.05) | –.03 (.07) |
| Task- \| Distraction | **–.23** (.07) | –.05 (.09) |
| Task- \| Social diversion | .02 (.05) | .03 (.07) |
| Emotion- \| Distraction | **.47** (.06) | **.58** (.08) |
| Emotion- \| Social diversion | **.30** (.05) | **.44** (.06) |
| Distraction \| Social diversion | **.94** (.03) | **1.06** (.08) |
| Well-being |  |  |
| Positive affect \| Negative affect | **–.31** (.04) | **–.23** (.06) |
| Positive affect \| Job satisfaction | **.36** (.05) | **.25** (.09) |
| Negative affect \| Job satisfaction | **–.22** (.05) | **–.29** (.08) |

*Note.* Reported are standardized parameter estimates with standard errors in parentheses.
Bold coefficients. *p* < .05 (one-tailed testing for directed hypotheses).

**Table S2**

*Standardized Estimates for Control Variables*

|  | T1 Coping strategies | | | |  | T1 Well-being | | |
| --- | --- | --- | --- | --- | --- | --- | --- | --- |
|  | Task-oriented | Emotion-oriented | Avoidance: Distraction | Avoidance: Social diversion |  | Positive affect | Negative affect | Job satisfaction |
| Gender | **–.11** (.05) | –.14 (.05) | **–.13** (.05) | **–.18** (.05) |  | **–.17** (.05) | **–.10** (.05) | **.11** (.05) |
| Rank: Predoc | –.03 (.06) | **.14** (.06) | .05 (.07) | .05 (.08) |  | **–.18** (.06) | .05 (.07) | **–.30** (.10) |
| Rank: Postdoc | .04 (.06) | .00 (.06) | .07 (.06) | **.12** (.05) |  | .06 (.07) | –.10 (.05) | **–.18** (.09) |
| General teaching experience | .08 (.07) | **–.15** (.06) | –.11 (.07) | **–.13** (.07) |  | –.07 (.06) | **–.13** (.06) | .09 (.07) |
| Online teaching experience | .00 (.05) | –.05 (.04) | –.07 (.05) | –.04 (.05) |  | –.00 (.04) | –.07 (.05) | .08 (.05) |
| Perceived impact of COVID-19 | –.04 (.05) | **.18** (.05) | **.14** (.05) | **.13** (.05) |  | **–.11** (.05) | **.32** (.04) | –.05 (.05) |
| Home office | .06 (.06) | .08 (.06) | .10 (.06) | .07 (.05) |  | –.01 (.05) | **.17** (.06) | –.01 (.05) |

*Note.* Reported are standardized parameter estimates with standard errors in parentheses. Bold coefficients. *p* < .05. Gender: 0 = *female*, 1 = *male*. Academic rank variables (predoc, postdoc): 0 = *no*, 1 = *yes*. Online teaching experience: 0 = *none*, 1 = *at least some*. General teaching experience: 0 = *less than 5 years*, 1 = *more than 5 years*. Perceived negative impact of COVID-19 pandemic on well-being (five-point Likert-scale): 1 = *not at all*, 5 = *very strongly*. Use of home office: 0 = *mostly working from home*, 1 = *mostly working in office*.
